# Assessment of magnetic flux density properties of electromagnetic noninvasive phrenic nerve stimulations for environmental safety in an ICU environment

**DOI:** 10.1038/s41598-021-95489-3

**Published:** 2021-08-11

**Authors:** K. Friedrich Kuhn, Julius J. Grunow, Pascal Leimer, Marco Lorenz, David Berger, Joerg C. Schefold, Steffen Weber-Carstens, Stefan J. Schaller

**Affiliations:** 1 Charité – Universitätsmedizin Berlin, corporate member of Freie Universität Berlin and Humboldt-Universität zu Berlin, Department of Anesthesiology and Operative Intensive Care Medicine , Charitéplatz 1, 10117 Berlin, Germany; 2 Switzerland Innovation Park Biel/Bienne, Biel/Bienne, Switzerland; 3 Universitätsklinik für Intensivmedizin, Inselspital, Universitätsspital Bern, University of Bern, Bern, Switzerland; 4 Technical University of Munich, School of Medicine, Department of Anesthesiology and Intensive Care, Ismaningerstr. 22, 81675 Munich, Germany

**Keywords:** Electronics, photonics and device physics, Medical research

## Abstract

Diaphragm weakness affects up to 60% of ventilated patients leading to muscle atrophy, reduction of muscle fiber force via muscle fiber injuries and prolonged weaning from mechanical ventilation. Electromagnetic stimulation of the phrenic nerve can induce contractions of the diaphragm and potentially prevent and treat loss of muscular function. Recommended safety distance of electromagnetic coils is 1 m. The aim of this study was to investigate the magnetic flux density in a typical intensive care unit (ICU) setting. Simulation of magnetic flux density generated by a butterfly coil was performed in a Berlin ICU training center with testing of potential disturbance and heating of medical equipment. Approximate safety distances to surrounding medical ICU equipment were additionally measured in an ICU training center in Bern. Magnetic flux density declined exponentially with advancing distance from the stimulation coil. Above a coil distance of 300 mm with stimulation of 100% power the signal could not be distinguished from the surrounding magnetic background noise. Electromagnetic stimulation of the phrenic nerve for diaphragm contraction in an intensive care unit setting seems to be safe and feasible from a technical point of view with a distance above 300 mm to ICU equipment from the stimulation coil.

## Introduction

Muscle atrophy, reduction of muscle fiber force and muscle fiber injuries are characteristics of ventilator-induced diaphragm dysfunction^1–3^. Mechanical ventilation applying positive pressure was shown to induce diaphragm muscle atrophy, loss of diaphragm strength, and adverse patient outcome in critically ill patients^4–8^. Diaphragm weakness affects approximately 60% of ventilated patients at the time of first spontaneous breathing trial^9–11^ and is twice as often as limb muscle weakness^10^. Disuse atrophy of the diaphragm can be observed within 12 h of mechanical ventilation and is the main culprit leading to ventilator-induced diaphragm dysfunction^1,3,6,7^. With up to 40% of critically ill patients on a medical intensive care unit (ICU) requiring mechanical ventilation, 20–25% may develop weaning problems^8,12,13^. Preserving diaphragm strength might be key to better weaning and improved patients outcome.

Electromagnetic stimulation of the phrenic nerve for diaphragm contraction and thus generating negative intrathoracic pressure might be an option for more physiological ventilation of critically ill patients requiring mechanical ventilation^4,14,15^. So far stimulation of the phrenic nerve was achieved using invasive techniques for electrical stimulation^2,16,17^.

Safety instructions recommend a minimum distance to magnetically-sensitive objects of 1 m and do not permit direct use on the heart. Further the use in patients with life-sustaining or supporting implants is contraindicated^18^. For that reason, we tested whether the use of electromagnetic stimulation is possible in an ICU setting to gain knowledge on patient’s safety when using electromagnetic stimulation of the phrenic nerve in patients with mechanical ventilation.

## Methods

We simulated a typical intensive care unit (ICU) setting to (1) measure distances between the patient and external medical equipment in a typical intensive care unit surrounding and (2) measure the magnetic flux density generated by the magnetic coils used for stimulation of the phrenic nerve. Since no patients were included in the study, there was no IRB approval necessary.

This simulation study was conducted twice at the Berliner Simulations- und Trainingszentrum at Charité—Universitätsmedizin Berlin in December 2019 and May 2020. For generalizability distance measurements were conducted at a second academic center (Inselspital Bern) in a typical ICU setting.

A typical ICU patient room was simulated and equipped with a Progressa bed (HILLROM BV, Amsterdam, The Netherlands), a Primus mechanical ventilator (Drägerwerk AG & Co. KGaA, Lübeck, Germany), an Injectomat MC Agilia (Fresenius Kabi Deutschland GmbH, Bad Homburg, Germany) and the PowerM Research 100 Stimulator (MAG & More GmbH, München, Germany) using a butterfly coil for stimulation (PMD70-pCool). For measuring of the magnetic flux density, a F3A Magnetic Transducer with fully integrated 1-/2-/3-axis hall probe (SENIS, Baar, Switzerland) was used. A Philips IntelliVue X2 monitor (Philips GmbH, Health Systems, Hamburg Germany) was used to investigate disturbances in electrocardiogram during stimulation. A resuscitation manikin was used to simulate the patient’s position in bed.

Stimulation of the phrenic nerve was performed at the muscle-free triangle at the neck just above the medial clavicula. To generate the highest possible magnetic flux density, a single biphasic stimulus at maximum intensity (100%) was used. Furthermore, a stimulation intensity of 40% was applied, as effectively used in earlier studies^4^. Magnetic flux density was measured at 0 mm, 20 mm, 30 mm, 70 mm, 300 mm and 400 mm vertical to the center of the coil. Furthermore, one measurement horizontal to the coil was done. Reliability between different measurements was assessed in a laboratory at the Switzerland Innovation Park Biel/Bienne.

In May 2020, we first measured the electromagnetic noise of the environment with all equipment turned off. Secondly, we measured the magnetic fields generated by ICU equipment only. Afterwards we measured the magnetic fields generated by the coil only. Lastly, we measured the magnetic fields with both, the coil and all ICU equipment switched on, to evaluate the combined fact.

Finally, we tested possible heating of metal surroundings and disturbances in electrocardiogram-monitoring (as e.g., known from the use of surgical bipolar tweezers). To evaluate heating of metal surroundings the stimulation coil was directly pointed at the bed gallow and temperature was measured with a FLIR SPOT TG165 infrared thermometer (FLIR Systems, Inc.). To trigger disturbances in electrocardiogram-monitoring a test person had to hold the electrocardiogram electrodes to enable electric conductivity. Then stimulation was applied.

## Results

### Distances

In our simulation the distance from the stimulation coil to the ventilator was 1.2 m. Limiting factor for maximum distance was the length of the breathing hose. The distance from the stimulation coil to the perfusor was 1 m. Further the distance from the stimulation coil to the touch panel of the bed was 0.7 m. Distances assessed in Bern were similar, i.e. approximately 0.8 m to the ventilator, 1 m to perfusors and to the vital signs monitor.

### Noise

The surrounding and intrinsic magnetic noise is presented in Additional File 1, Fig. S1. Analyzing the frequency spectrum, a dominant frequency at 16,019 Hz can be observed at 250 mm above the coil. It is not found at 1 m above the coil (see Additional File 1, Fig. S2). With only ICU equipment switched on, the only signal detectable is at 1 m above the coil. For all other distances, the signal is below noise level (< 1 mT, see Additional File 1, Fig. S3a). With only the stimulator switched on and all other equipment switched off, a typical magnetic field response can be measured up to a distances of 200 mm above the coil. In greater distances the signal cannot be distinguished from background noise. For distances up to 100 mm above the coil, a dominant frequency at around 6 kHz is observed, corresponding to a pulse length of 0.166 ms (see Additional File 1, Fig. S3b).

At both, ICU equipment and stimulator switched on, typical magnetic field responses can be measured up to 200 mm above the coil. Notably peaks at around 3 mT were detectable in greater distances (300 mm to 1000 mm). The frequency spectrum corresponds with the frequency spectrum when ICU devices are switched off (see Additional File 1, Fig. S3c).

### Magnetic flux density

**Figure 1:**
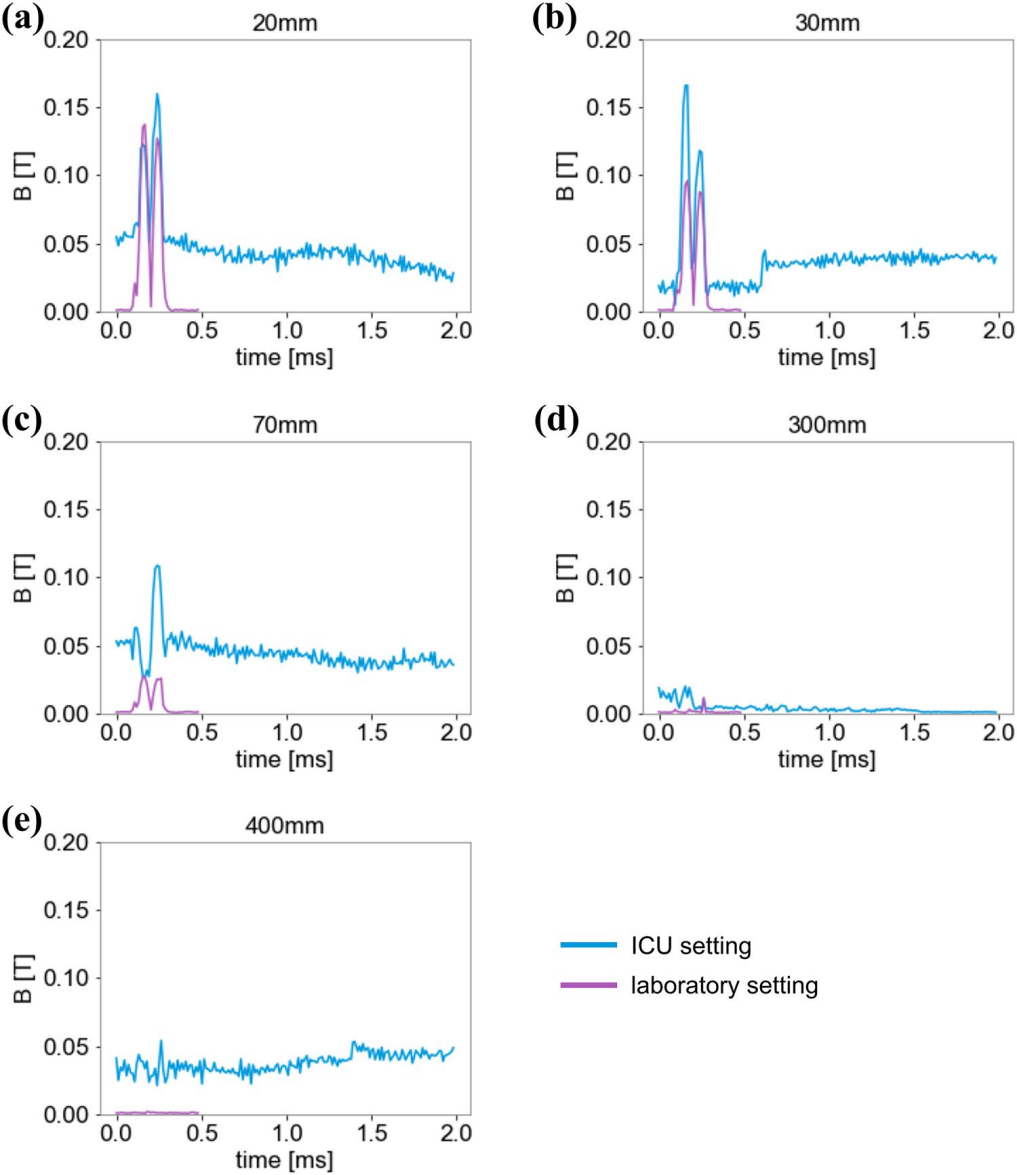
Magnetic flux density at different distances vertical to the coil. Measurements at different distances to the stimulation coil were done. At 300 mm and 400 mm a lot of artefacts occurred and no clear signal was detectable. Results in the ICU setting in general show a lot of noise, offset and amplification due to surrounding electronic devices.

**Figure 2:**
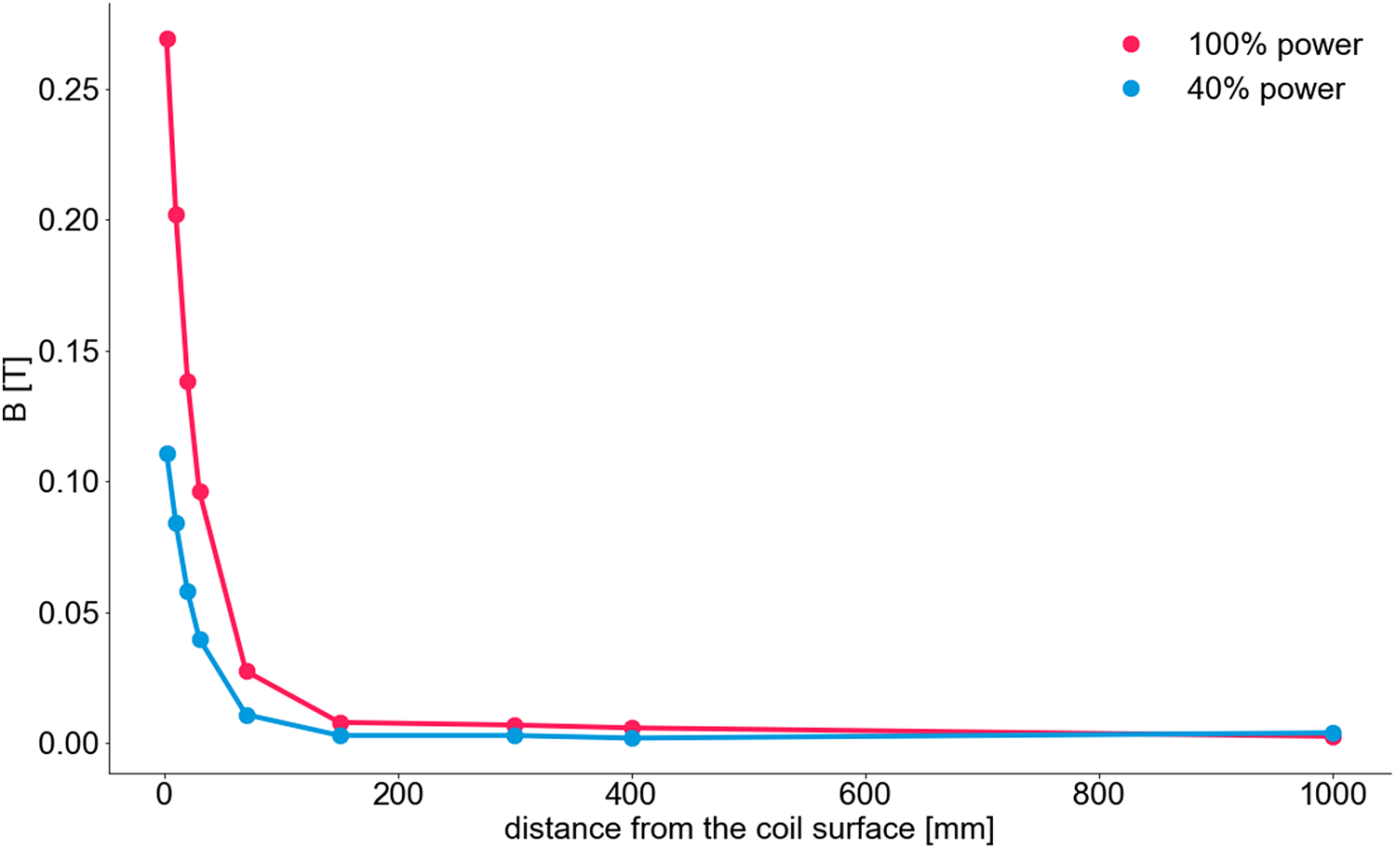
Magnetic flux density at different test points. Measurement of magnetic flux density at different test points in laboratory. Magnetic flux density showed exponential decline with distance from the coil surface. Measurements were done at 100% and 40% power. Points are connected with lines for visual guidance. *B* magnetic flux density (Tesla).

**Figure 3:**
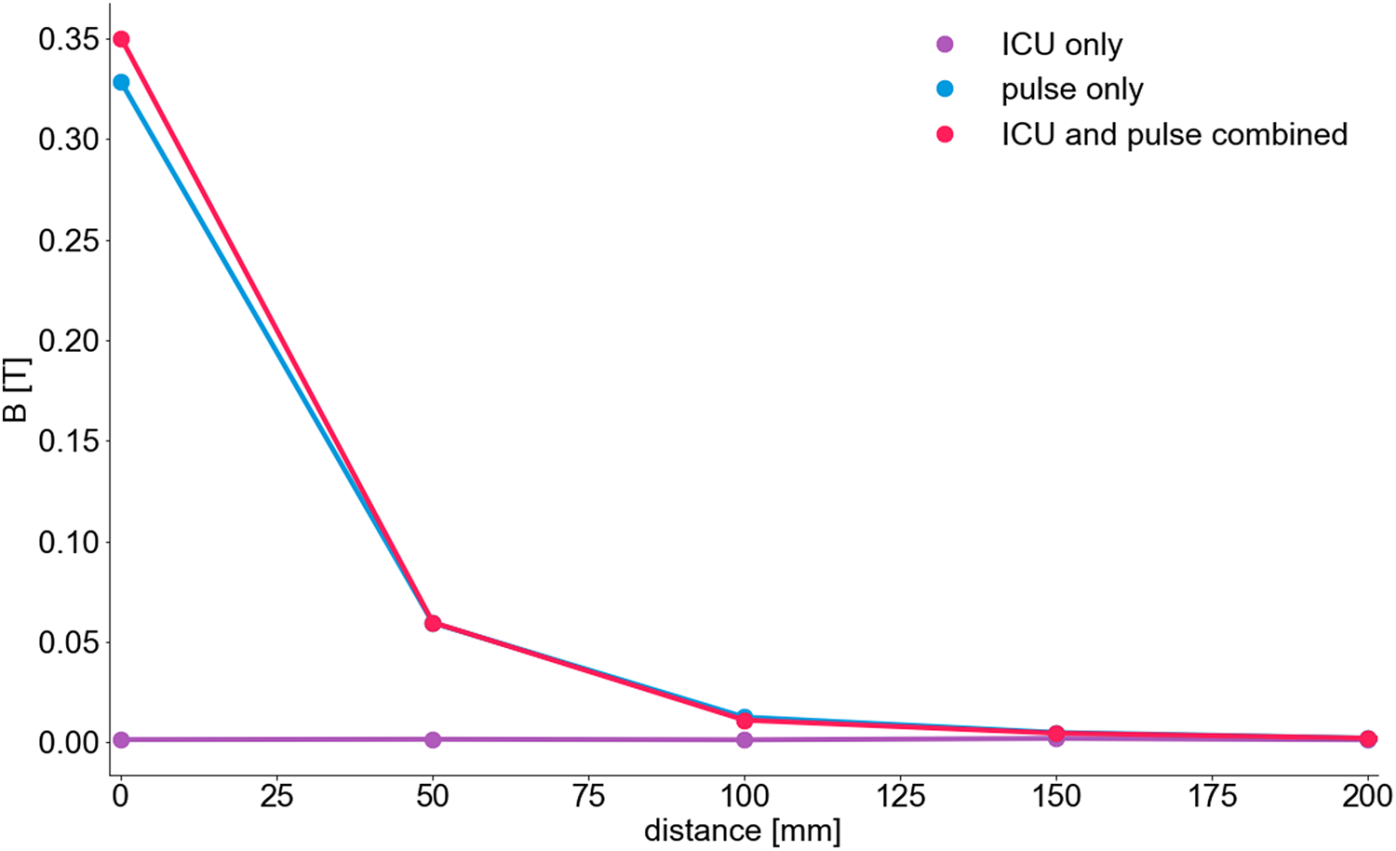
Magnetic flux density in ICU setting at different test points. Measurement of the magnetic flux density was done at different test points in the ICU with only ICU equipment switched on, only stimulator switched on and ICU and stimulator switch on at the same time. Points are connected with lines for visual guidance. *B* magnetic flux density (Tesla).

There was a high reliability between measurements of the magnetic flux density (see Additional File 1, Fig. S4). At 100% power of the device the magnetic flux density at the center of the coil was 1000 mT (Table 1). With rising distance to the coil, the magnetic flux density declined exponentially. At a distance of 300 mm the magnetic flux density was 30 mT in the ICU setting and lower (7 mT) under laboratory conditions. At 300 mm and 400 mm, however, the signal cannot be distinguished from the surrounding and intrinsic magnetic noise (Fig. 1). Results and exponential decline were similar at 40% intensity and 100% intensity with single, biphasic impulse (Fig. 2). The magnetic flux density showed similar decline with rising distance from the stimulation coil with all ICU equipment switched off as well as with ICU equipment switched on (Fig. 3).

**Table 1:** Comparison of magnetic flux density in a laboratory and ICU setting at different distances to the stimulation coil.

Distance (mm)	Stimulus	Magnetic flux density B (T)		
		laboratory setting		ICU setting
		40% Power	100% Power	100% Power
0	Single	n.a.	n.a.	1
2	Single	0.111	0.269	n.a.
10	Single	0.084	0.202	n.a.
20	Single	0.058	0.138	0.3
30	Single	0.040	0.096	0.2
70	Single	0.011	0.028	0.1
150	Single	0.003	0.008	0.03
300	Single	0.003	0.007	0.05
400	Single	0.002	0.006	0.03
1000	Single	0.004	0.003	n.a

*B (T)* magnetic flux density in Tesla.

### Heating and disturbances

With 85% intensity, a frequency of 20 Hz and 2 to 3 sequences with a length of 5 to 10 s the bed gallow heated to 70 °C at direct contact between the coil and the gallow. Simultaneously, the stimulation coil quickly heated up to the maximum temperature of 40 °C leading to a safety stop. At a distance of 5 cm to the gallow slight warming was detectable after 5–10 s of stimulation.

The electrocardiogram electrodes did not heat up. The electrocardiogram signal at the monitor showed artefacts during stimulation as known from surgical bipolar tweezers.

All other surrounding medical equipment as described above did not show any malfunction as well as direct stimulation of the perfusors.

## Discussion

In this simulation study we measured magnetic flux densities at different distances to a stimulation coil to gain knowledge on possible interferences with electronic equipment in an ICU setting. Magnetic flux density showed an exponentially decline with distance to the coil with no detectable signal distinguishable from surrounding noise above 300 mm.

We did not find any information on maximum magnetic flux densities safe to use near ventilators apart from general warnings that portable and mobile radiofrequency communications equipment can affect ventilators and that special precautions regarding electromagnetic compatibility are mandatory^19,20^. Regulations of magnetic stimulation devices embrace to not use the stimulation coils closer than 1 m to any sensitive electronics^21,22^. Of note these regulations are designed for transcranial magnetic stimulation, the most common clinical use. However, in a non-ICU setting of repetitive transcranial magnetic stimulation in 30 pregnant women with depression a safety distance below 1 m was safely used^23^. Furthermore, repetitive transcranial stimulation was well tolerated and the subsequent follow-up study on the children of these women did not show poorer cognitive or motor development^24^.

In an ICU setting, however, the major concern is interference of the electromagnetic field with life-sustaining equipment. Only three studies were investigating magnetic stimulation in critically ill patients, two in patients with refractory status epilepticus, one to assess diaphragm weakness^10,25–27^. Liu et al. applied 70% output when using repetitive transcranial magnetic stimulation for refractory focal status epilepticus in intubated and sedated patients. To avoid any disruptions of sensitive medical equipment they repeatedly discharged their butterfly coil at 100% output in midair without any interference. In that study, the following equipment was not harmed during stimulation: clinical computer console, vital signs monitor, ventilator, medication pump, feeding pump, bed, pneumatic boot machine, and electroencephalogram^26^. Thordstein et al. applied 60 min of stimulation with 0.5 Hz at a 68-year old patient with refractory partial status epilepticus using a butterfly coil as well^25^. In the third study using bilateral magnetic phrenic nerve stimulation to assess diaphragmatic dysfunction in 76 mechanically ventilated patients two figure-of-eight coils were used. A maximum stimulator output (100%) was applied^10^. In all three studies no adverse events related to the stimulation were reported. No interference with ICU equipment and monitoring was reported in all three ICU studies, however, there was no description of distances of the ICU equipment and the stimulation coil. Our study is the first to investigate the distances between the stimulation coil and the surrounding equipment as well as the magnetic flux decline.

As there are only few studies using electromagnetic stimulation in ICU, patients’ safety remains an issue. Current *Guidelines of the International Commission on Non-Ionizing Radiation Protection* describe any magnetic flux density up to 0.1 mT as not harmful for human beings^28^. Our results at 100% power show these 0.1 mT are reached at a distance of 1 m to the coil. To further address safety measures dosimetry can be used to determine the induced electrical field. However, the current *European Respiratory Society statement on Respiratory Muscle Testing at Rest and during Exercise* declares that it is possible to assess diaphragmatic contractility in critically ill, intubated patients by magnetic stimulation of the phrenic nerve^29^. In a study using electromagnetic stimulation of the phrenic nerve in 10 healthy probands no safety issues were reported^4^. 6 volunteers reported dental paresthesia during stimulation which can be confirmed by our own observations. They used 20%, 30% and 40% output of the stimulator which was well tolerated. Using more output led to strong muscle activation and discomfort in probands. Adler et al. also used stimulation of the phrenic nerve for diaphragm contraction in 7 healthy probands^30^. They also did not report any safety issues. Tolerance by test persons was good though with increasing intensity discomfort was also increasing.

The generalizability of our study may be limited because we only measured distances in two ICU settings, however, both were quite similar. Secondly, we are only able to make statements of missing disturbances for the equipment mentioned above. Since there was no signal discriminable from background noise above 300 mm, a safety distance above that range should be safe independent of the ICU equipment manufacturer.

In conclusion, electromagnetic stimulation seems possible in an ICU environment with adequate safety distance. At distances above 300 mm only background noise could be measured.

## Supplementary Information

Supplementary Information.

## Data Availability

The datasets generated during and/or analysed during the current study are available from the corresponding author on reasonable request.
