# Supplementary material for: Assessment of magnetic flux density properties of electromagnetic noninvasive phrenic nerve stimulations for environmental safety in an ICU environment
Source: Sci Rep. 2021 Aug 11;11:16317. doi: 10.1038/s41598-021-95489-3 (PMC8357944; doi:10.1038/s41598-021-95489-3)
Supplement: Supplementary file 1 — Supplementary Information. [file 41598_2021_95489_MOESM1_ESM.pdf]

Additional file 1

Assessment of magnetic flux density properties of electromagnetic noninvasive phrenic nerve stimulations for environmental safety in an ICU environment

Figures

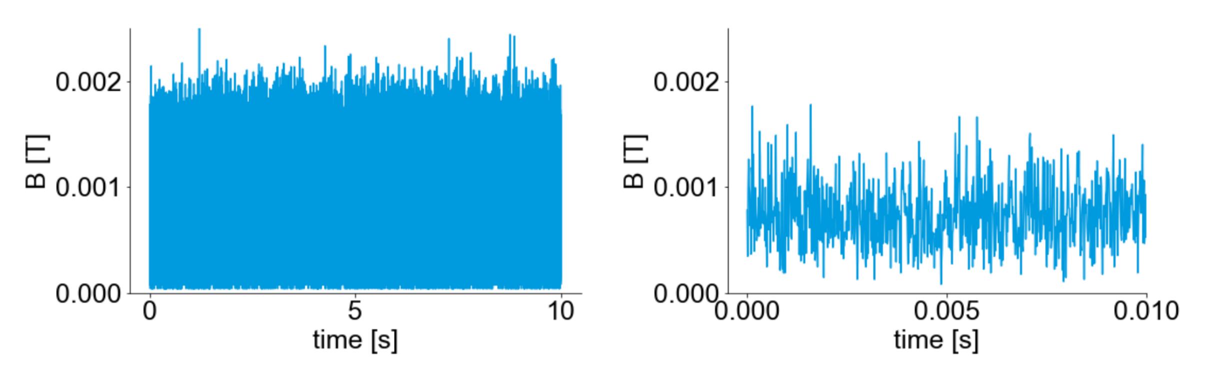

Figure S1. Magnetic flux density with all devices switched off.

Left: Measured magnetic flux density during 10 s with ICU equipment and stimulator switched off. Right: Same data but limited to the time range 0-0.01 s. B = magnetic flux density [Tesla].

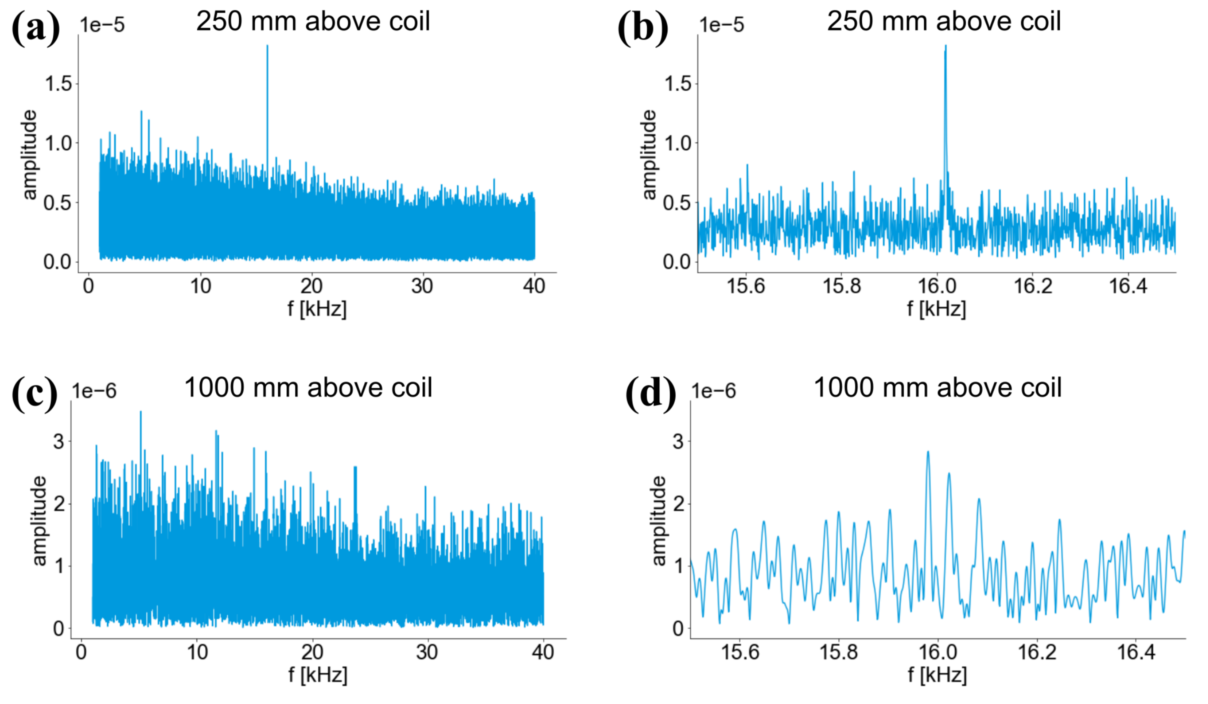

Figure S2. Noise level above the stimulation coil.

Left: Frequency spectrum of noise 250 mm and 1000 mm above the coil. Right: Same data but limited to the frequency range 15.5-16.5 kHz. B = magnetic flux density [Tesla].

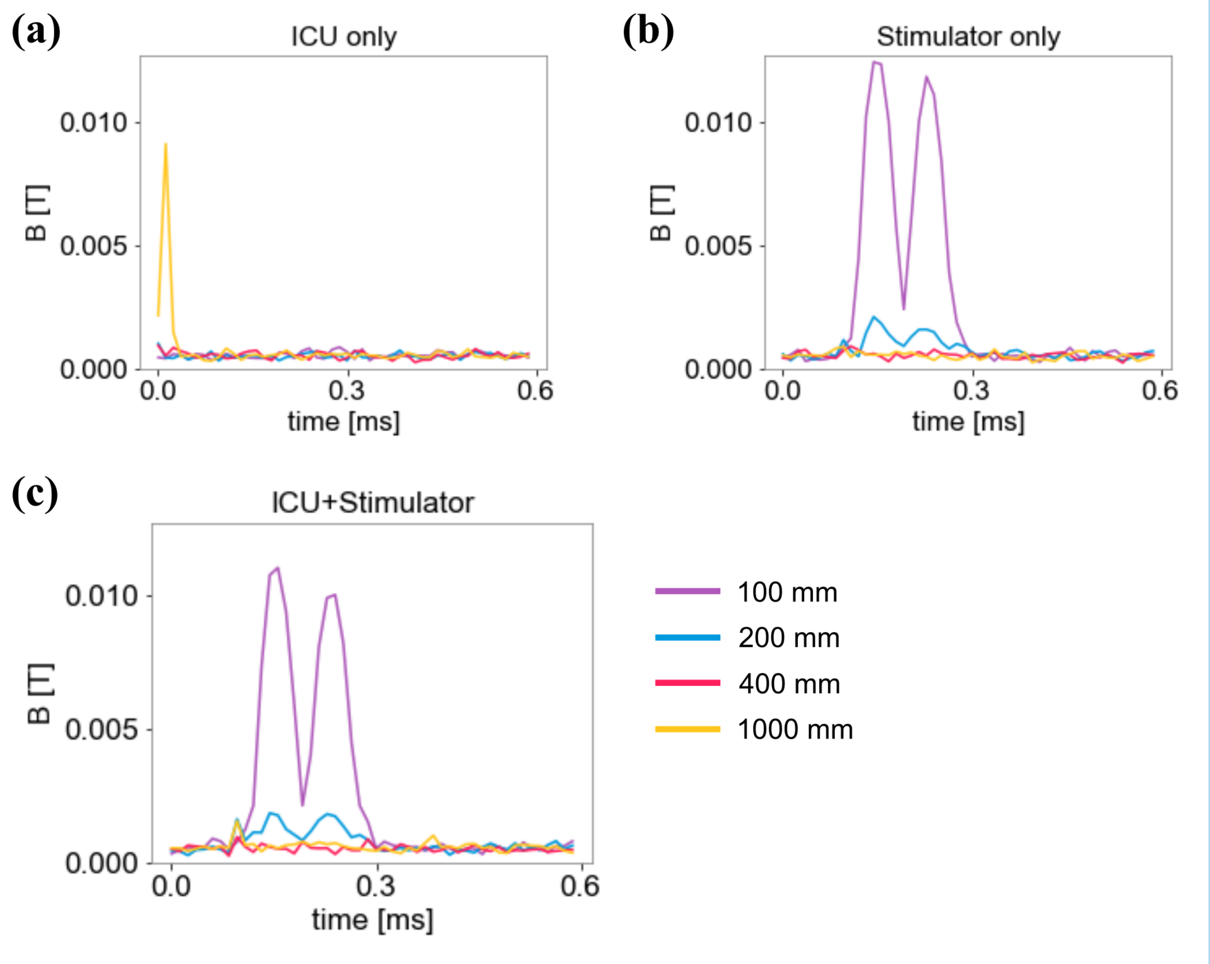

Figure S3. Magnetic flux density at different distances above the stimulation coil.

Magnetic flux density at 100 mm, 200 mm, 400 mm and 1000 mm above the coil with only ICU equipment switched on (a), only the stimulation switched on (b) and ICU equipment and stimulator switched on (c).

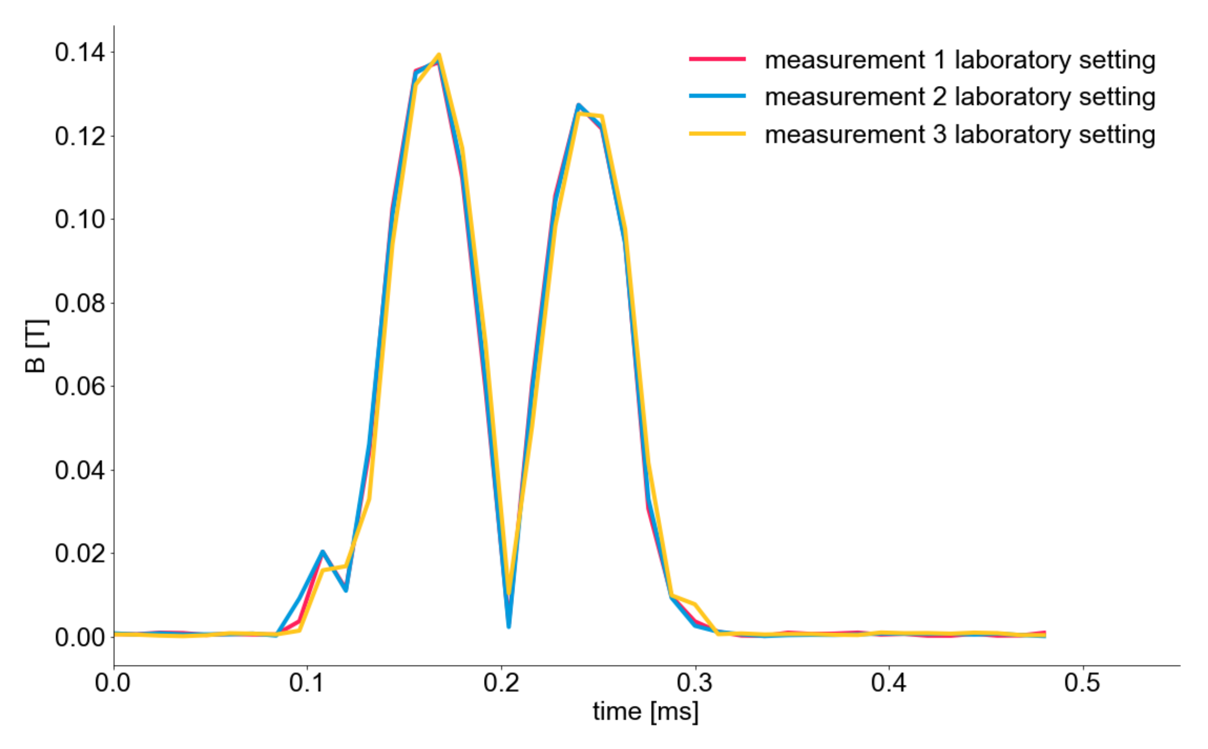

Figure S4. Reliability between measurements.

A single biphasic pulse was applied in laboratory for three times to determine reliability between measurements. The pulse length was 0.1 ms with a maximum magnetic flux density of 0.14 Tesla. B = magnetic flux density [Tesla].
